# Preparation of cross-linked hen-egg white lysozyme crystals free of cracks

**DOI:** 10.1038/srep34770

**Published:** 2016-10-05

**Authors:** Er-Kai Yan, Qin-Qin Lu, Chen-Yan Zhang, Ya-Li Liu, Jin He, Da Chen, Bo Wang, Ren-Bin Zhou, Ping Wu, Da-Chuan Yin

**Affiliations:** 1 Institute for Special Environmental Biophysics, Key Laboratory for Space Bioscience and Biotechnology, School of Life Sciences, Northwestern Polytechnical University, Xi’an 710072, Shaanxi, P. R. China

## Abstract

Cross-linked protein crystals (CLPCs) are very useful materials in applications such as biosensors, catalysis, and X-ray crystallography. Hence, preparation of CLPCs is an important research direction. During the preparation of CLPCs, an often encountered problem is that cracks may appear in the crystals, which may finally lead to shattering of the crystals into small pieces and cause problem in practical applications. To avoid cross-link induced cracking, it is necessary to study the cracking phenomenon in the preparation process. In this paper, we present an investigation on how to avoid cracking during preparation of CLPCs. An orthogonal experiment was designed to study the phenomenon of cross-link induced cracking of hen-egg white lysozyme (HEWL) crystals against five parameters (temperature, solution pH, crystal growth time, glutaraldehyde concentration, and cross-linking time). The experimental results showed that, the solution pH and crystal growth time can significantly affect cross-link induced cracking. The possible mechanism was studied, and optimized conditions for obtaining crack-free CLPCs were obtained and experimentally verified.

It is well-known that protein crystals are fragile and hard to handle. Due to their bad mechanical properties, the application of protein crystals is restricted to limited fields. So far, a number of methods have been developed to obtain high quality protein crystals^1^,^2^,^3^,^4^,^5^,^6^,^7^,^8^. However, these methods cannot obtain mechanically stable protein crystals. In order to widen their applications, it is necessary to ameliorate the mechanical stability of the crystals. One way to solve this problem is utilisation of chemical cross-linking of protein crystals using glutaraldehyde, which is an efficient way to confine protein molecules in the crystal lattice and hence greatly improve crystal stability^9^. There are strong covalent bonds formed after cross-linking by glutaraldehyde (including intermolecular cross-links and intramolecular cross-links)^10^, leading to consolidation of the crystal structure. Cross-linked protein crystals are insoluble in water and organic solvents^11^, and can be directly handled by mechanical contact. Moreover, their mechanical stability^12^, thermal stability^13^, and activity in organic solvents^11^ are significantly enhanced after being treated with glutaraldehyde. The viscoelastic properties^14^, composition and mobility of ions, ionic conductivity, transference numbers of cross-linked lysozyme crystals^15^ and their permeability to water^16^ were also improved. Such excellent properties of CLPCs make them potentially useful in the fields of biosensors^17^, synthetic chemistry^18^, materials science^19^, oral delivery^20^, and chromatographic analysis^21^, etc. In addition, chemical cross-linking via glutaraldehyde may also improve the quality of protein crystals^22^,^23^,^24^, which is essential for their use in high resolution structural determination.

Many kinds of cross-linked protein crystals, such as cross-linked glucose isomerase crystals^25^ and thermolysin crystals^26^, had been prepared and studied. These CLPCs are indeed potentially useful; however, a problem is often encountered, that may hinder their practical application. That is, cross-linking induces cracking of the crystals, which is an often occurring but seldom addressed issue. This issue has not been fully studied, and methods of solving the problem remain to be explored.

To find solutions to this problem, we initiated an investigation into the phenomenon, i.e., cross-link induced cracking of protein crystals, under different preparation conditions using an orthogonal experiment design. Preparation parameters, i.e., crystal growth time, temperature, cross-linking time, solution pH, and glutaraldehyde concentration, were used as factors, and hen-egg white lysozyme (HEWL) was used as the model protein. The major goal was to find the effects of the preparation parameters on the cracking ratio, and finally, based on the results, propose suitable conditions for preparing cross-linked lysozyme crystals free of cracks.

## Results and Discussions

### Morphology of the crystals

**Figure 1:**
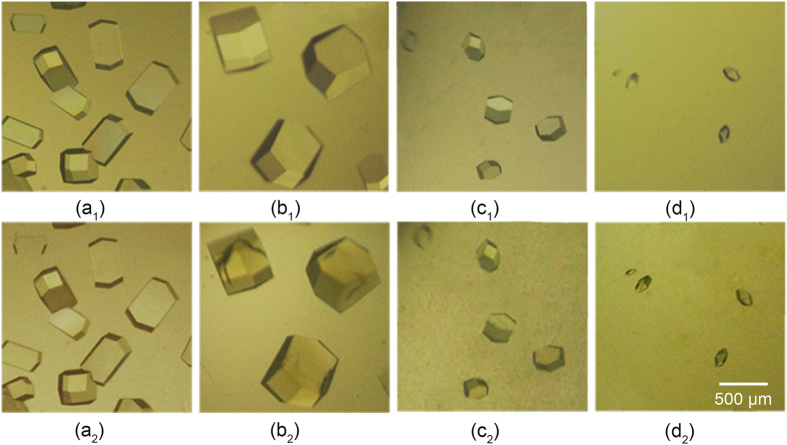
Typical examples of crystals morphology before and after chemical cross-linking at 17 °C. (**a**) 5 days, pH = 4.2; (**b**) 4 days, pH = 5.4; (**c**) 3 days, pH = 6.6; (**d**) 2 days, pH = 7.8. The subscripts 1 and 2 indicate the crystal images before and after cross-linking, respectively.

Images of the HEWL crystals were captured before and after cross-linking. Figure 1 shows several examples at different conditions. From the images in the upper row (Fig. 1(a_1_–d_1_)), it can be seen that the crystal morphology evolved from rectangular-shaped to square-shaped and finally to diamond-shaped upon changing the pH from 4.2 to 7.8. Such shape variation against pH is in good accordance with results reported in the literature^27^. It was found that the area of lysozyme crystals reached maximum at pH = 5.4. The lower row (Fig. 1(a_2_–d_2_)) shows images of the crystals after cross-linking via glutaraldehyde. It can be seen that, some crystals showed obvious cracks on the surface (as seen in Fig. 1(b_2_–d_2_)). The number of cracks in the crystals increased with increasing cross-linking time. The lysozyme crystals growing from the mother liquid were clear and colourless, while those after cross-linking showed a pale yellow colour, and the colour would gradually darken with increasing cross-linking time.

### Ratio of cracked CLPCs

**Figure 2:**
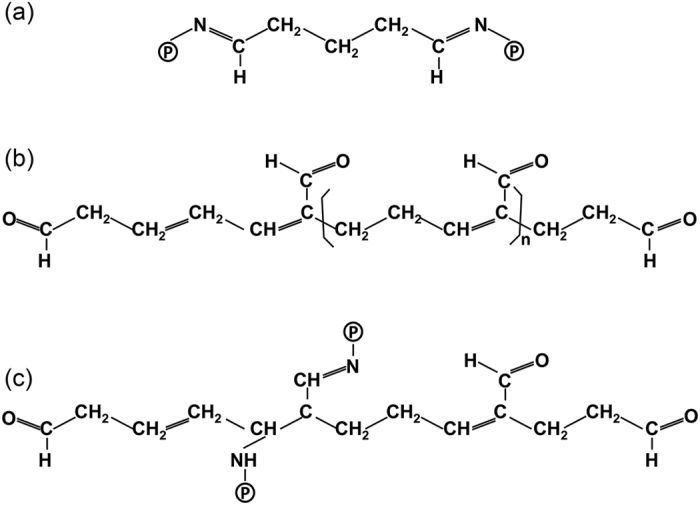
Schematic representation of the chemical cross-linking. (**a**) Schiff base formation in the cross-linking between lysine residues from two protein molecules and monomeric glutaraldehyde, (**b)** Structure of polymeric glutaraldehyde, (**c)** Suggested end product formation obtained by polymeric glutaraldehyde and lysine residues from the cross-linked proteins^28^.

In the current study, the reactions between lysine groups in CLPCs and aldehyde molecules in glutaraldehyde are the dominant reaction during the cross-linking process^28^, as shown in Fig. 2. Apart from the dominant reactions, there are also reactions between glutaraldehyde (or its polymerisation products in glutaraldehyde) and other enzyme moieties, such as amines, thiols, and imidazoles^29^. These multiple reactions make the reaction between the glutaraldehyde and protein crystal more complicated, and the reaction would continue if not stopped. During cross-linking, new chemical bonds will form between the glutaraldehyde molecules and protein molecules. These bonds contribute to maintaining the highly ordered three-dimensional (3D) arrangements of protein molecules. However, the formation of the new chemical bonds can also result in an increase of internal stress, which will destroy the original lattice structure, and cause cracking of the crystals.

Obtaining cross-linked crystals free of cracks is the major goal of this research. Hence we used the ratio of cracked CLPCs (we call it crack ratio in this work, and it is defined as the ratio of the cracked crystal number to the total crystal number) as a target parameter. Cracked crystals are defined as those showing obvious cracks in the crystal image under the stereomicroscope at a magnification of 50×.

The data on crack ratio, and crystal area of the CLPC in this study are summarised in Table 1. All of the data (crack ratio and crystal area) of hundreds of crystals were analysed using a statistical analysis from the 15 repeated experiments, and the results are listed in Table 1. We noticed that the HEWL crystals did not crack at all at pH = 4.2, which is a desirable condition. However, the crystal area growing at pH = 4.2 was relatively small. In addition, we observed that combination 6 (the Experimental run No. 6) corresponds to the largest crystals area while the crack ratio was extremely low. The conditions were temperature 13 °C, pH = 5.4, crystal growth time 2 days, glutaraldehyde concentration 4%, and cross-linking time 3 days.

### Intuitive analysis

**Figure 3:**
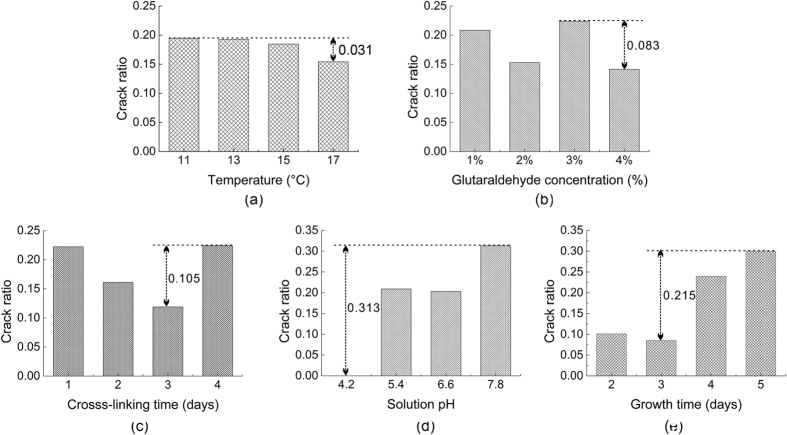
Visual analysis chart of the influence of various factors on crack ratio of crystals in the orthogonal experiment. (**a**) Temperature; (**b**) Glutaraldehyde concentration; (**c**) Cross-linking time; (**d**) Solution pH; (**e**) Growth time. Note: Every column in the figure represents the averaged crack ratio of four combinations in the orthogonal array, and there is no error bar.

According to the experimental data, we conducted an intuitive analysis to find out the primary and secondary factors affecting the crack ratio (Fig. 3). The ordinate represents the crack ratio and the abscissa represents the factors (temperature, glutaraldehyde concentration, cross-linking time, solution pH and crystal growth time). Every column in the figure represents the averaged crack ratio of four combinations in the orthogonal array. The range of each factor is marked in the figure. Among the five factors, solution pH and crystal growth time correspond to the largest range, indicating that these two factors can exert the most significant impact on cross-linking induced cracking. The remaining three factors correspond to a smaller range, indicating that these three factors have less effect on cross-linking induced cracking.

According to Fig. 3(a), the range of the crack ratio under different temperatures was only 0.031, which means the crack ratio was not sensitive to temperature. In our current study, the temperature range was from 11 to 17 °C, which is a relatively suitable range for the growth and cross-linking reaction of lysozyme crystals. Hence we did not observe any striking effect of temperature on the crack ratio.

According to Fig. 3(b,c), the crack ratio ranges across glutaraldehyde concentration and cross-linking time were 0.083 and 0.105, respectively, indicating that both glutaradehyde concentration and cross-linking time had an impact on crystal cracking to some extent. However, the influence was not significant, and there was no obvious trend.

As shown in Fig. 3(d), solution pH had the largest crack ratio range, which means it had the most significant effect on the crack ratio. The crack ratio increased as the pH increased, with the lowest crack ratio being found at pH = 4.2, and the largest crack ratio at pH = 7.8 in the studied pH range. There are several factors that may contribute to the phenomenon. The cross-linking process using glutaraldehyde mainly includes two reactions: the self-polymerization of glutaraldehyde and the covalent cross-linking between protein molecules and aldehyde molecules. The properties of glutaraldehyde as a cross-linking agent change greatly under an acidic or alkaline environment, especially its self-polymerization reaction^18^,^30^,^31^. Glutaraldehyde exhibits stability and its polymerization is relatively low in acidic conditions^32^. In addition, acidic conditions are far from the isoelectric point of HEWL (pI = 11.3), which makes acidic conditions more suitable for the growth of lysozyme crystals, and contributes to obtain more regular crystal structure with less lattice imperfection. The perfect lattice structure of crystals and the stable nature of glutaraldehyde may lead to a lower crack ratio under acidic conditions than alkaline conditions. In contrast, glutaraldehyde exhibits high self-polymerization^33^,^34^ and produces a mixture with different structures and lengths^35^,^36^ in alkaline conditions. The cross-linking reactions between such a mixture and crystals are not limited to one reaction and would yield complicated products. Such intricate reactions and products make the cross-linking inhomogenous both inside and outside of crystals, cause internal stress inside CLPCs and then induce the crystal to crack. In addition, alkaline conditions are not very suitable for the growth of high quality lysozyme crystals. Furthermore, we found the crack ratio of crystals was 0.2027 using aged (highly polymerized) glutaraldehyde solution, while the crack ratio of crystals was only 0.0769 using freshly prepared (less polymerized) glutaraldehyde solution. The result showed that the condition of the glutaraldehyde solution (aged or freshly prepared) can also affect the crack ratio of the crystals, probably due to the self-polymerization of glutaraldehyde in the aged solution. On the whole, the different polymerization degrees of glutaraldehyde and the more comfortable crystal growth environment may be important reasons for the observed phenomenon.

As for the effect of crystal growth time on the crack ratio, it may be easy to understand. Longer growth time under the same crystallization conditions usually results in larger crystal area, which may lead to more stacking defects and dislocations due to the (internal) stress in the crystal lattice, and hence it will be easier to cause cracking of the crystals. In addition, the dislocations play an even more important role. Furthermore, the cross-linking reaction is a process occurring from outside to inside of the crystals. It is very difficult to cross-link crystals homogeneously from outside to inside because homogeneous mass-transport of glutaraldehyde is hard to achieve throughout the whole crystal due to the transport path being from outside to inside. Such an inhomogeneous cross-linking process throughout the crystal will result in inhomogeneous distribution of cross-linking products in the crystals and hence cause internal stress, which will finally results in cracking of the crystal.

**Figure 4:**
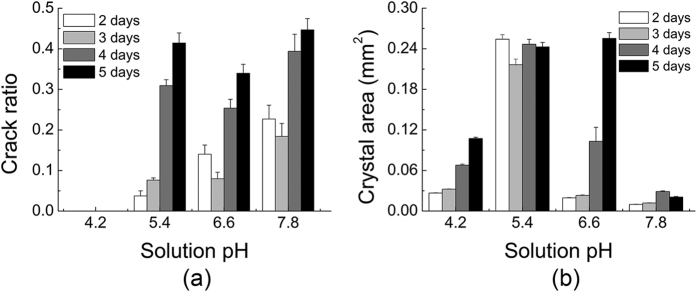
>Crack ratio, and crystal area of protein crystals growing for 2, 3, 4, or 5 days at pH values of 4.2, 5.4, 6.6, or 7.8. (**a**) Crack ratio. (**b**) Crystal area.

In order to further understand the mechanism linking the crack ratio and crystal growth time, the crack ratio and crystal area of crystals growing for 2, 3, 4, and 5 days at pH values of 4.2, 5.4, 6.6, and 7.8 were plotted in Fig. 4. From Fig. 4(a), we can observe that the crack ratio increased with increasing growth time at the same pH. In light of this phenomenon, we analysed the relationship between crystal area and growth time, and the results are shown in Fig. 4(b). As expected, the crystal area increased with growth time. However, there were some exceptions; for example, when pH = 5.4, it seems that the crystal area decreased with growth time. However, due to some reasons such a phenomenon may occur: (1) the measurement of crystal area is only an approximate method, crystal area is only a two-dimensional parameter, which cannot truly represent the three-dimensional area; (2) the crystallisation process of protein often suffers from bad reproducibility, hence the results may deviate from the expected ones; (3) the actual growth conditions were not identical because we used an orthogonal array to carry out the crystallisation experiment, and each combination is different from the others even though some parameters are identical like solution pH.

Figure 4(b,c) describes the crystal area of HEWL crystals growing for different time against the pH of the crystallisation solution. When comparing the crack ratio among different pH groups, we cannot reach the conclusion that the larger the crystal is, the higher the crack ratio will be. An obvious exception is the case when pH = 7.8, in which condition the crystal area was very small; however, the crack ratio was rather large; whereas when pH = 4.2, all crystals with different areas did not crack, verifying that the pH is a very essential parameter for controlling the crack ratio.

**Figure 5:**
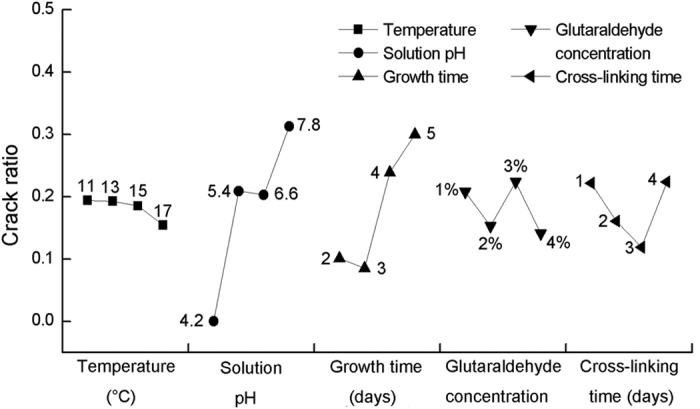
Curve diagram of crack ratio for the orthogonal experiment. The abscissa corresponds to different levels of each factor, including temperature, pH, growth time, glutaraldehyde concentration, and cross-linking time.

In order to identify any trends between the influencing factors and the crack ratio more intuitively, a curve diagram (Fig. 5) was drawn to screen appropriate cross-linking conditions. From Fig. 5, we can easily find the optimum conditions at temperature 17 °C, pH = 4.2, growth time 3 days, glutaraldehyde concentration 4%, and cross-linking time 3 days when not considering crystal area (optimum condition 1). The crack ratio was 0 at pH = 4.2, and another low crack ratio was 3.78% at combination 6 (optimum condition 2) in Table 2. These two combinations correspond to the same glutaraldehyde concentration (4%) and cross-linking time (3 days), while the other three conditions (temperature, solution pH and crystal growth time) were not the same. Intuitive analysis shows that the influence of temperature on the cracking of crystals was small, and the difference in temperature between the two optimum conditions was acceptable. Differences in solution pH and crystal growth time contribute to the growth of large scale crystals. If obtaining completely perfect CLPCs are desired, optimum condition 1 would be the optimal choice; whereas if large CLPCs with few imperfections are needed, optimum condition 2 would be the optimal choice.

Finally, we carried out a verification experiment based on optimum condition 1, and the results showed that the crack ratio of cross-linked HEWL crystals was indeed the best (i.e., crack ratio = 0). In addition, we performed a cross-linking experiment with lysozyme crystals growing at pH = 3.6, with other conditions (temperature, growth time, glutaraldehyde concentration and cross-linking time) being the same as optimum condition 1. The results showed that the crack ratio of the crystals (not cross-linked yet) was high, and the perfect crystals did not crack after cross-linking by glutaraldehyde. Furthermore, the crystals were rod-like, and their area was larger than those growing at pH = 4.2.

### Multi-factor analysis of variance

Visual analysis methodology can effectively determine the primary and secondary factors effecting crystal cracking, but it cannot distinguish the data fluctuations caused by the experimental conditions or by experimental error. Therefore, we carried multi-factor analysis of variance (Table 2) using F tests to analyse the significance of each factor. The freedom degree of the factors was 3. We chose the reaction temperature as the reference, whose square of deviance (DEVSQ) was the lowest. The results showed that the solution pH and crystal growth time indeed significantly affected the cross-linking reaction between lysozyme crystals and glutaraldehyde (P < 0.01). The cross-linking time showed a certain effect on the cross-linking reaction (P < 0.1). The results of the multivariate analysis of variance are highly consistent with the visual analysis, namely differences in crack ratio primarily derive from the solution pH and crystal growth time, followed by cross-linking time.

### The diffraction analysis

In order to further explore the effect of cross-linking reaction on the protein crystals, the diffraction patters of crystals were analysed. X-ray diffractometer (Mar μX, Mar research, Germany) was used to obtain the diffraction data of native crystals and CLPCs, and the HKL 2000 packages were used to integrate and analysis the diffraction data. The CLPCs (optimum condition 1) diffracted to 1.83 Å using the diffractometer, which was analogously with the native crystals (1.69 Å) using the same diffractometer. And the mosaicity decreased from 0.97 to 0.77 after cross-linking by glutaraldehyde. On the whole, there are only minor differences in the diffraction pattern before and after cross-linking, which is in accordance with the reported literatures^37^,^38^,^39^.

## Concluding Remarks

In the present study, we used an orthogonal array to investigate the cracking of lysozyme crystals cross-linked by glutaraldehyde. A large number of experiments were performed to examine the factors affecting the cracking of the crystals, aiming to provide guidance for cross-linking lysozyme crystals free of cracks and to lay the foundation for practical applications. According to the results presented in this paper, the solution pH showed the most significant effect on the cross-linking induced cracking. Crystal growth time showed the second strongest effect, followed by cross-linking time in the orthogonal experiment. Both visual analysis and variance analysis confirmed the above results. The optimized cross-linking conditions for obtaining lysozyme crystals free of cracks were: temperature 17 °C, pH = 4.2, growth time 3 days, glutaraldehyde concentration 4%, and cross-linking time 3 days when not considering crystal area. If obtaining large cross-linked lysozyme crystals is desired, the optimized cross-linking conditions should be: temperature 13 °C, pH = 5.4, growth time 2 days, glutaraldehyde concentration 4%, cross-linking time 3 days.

## Materials and Methods

### Materials

Hen egg white lysozyme (HEWL; catalogue No. 100940, six times recrystallised, Seikagaku Corp., Japan) was purchased and utilised without further purification. Other chemical reagents utilised include: sodium chloride (NaCl, Chemical Reagent Co. Ltd, China), glutaraldehyde (Chemical Reagent Co. Ltd, China), sodium acetate (CH_3_COONa•H_2_O, Xi’an Chemical Factory, China), sodium dihydrogen phosphate (NaH_2_PO_4_•2H_2_O, Xi’an Chemical Factory, China), acetic acid (CH_3_COOH, Beijing Chemical Factory, China), and disodium hydrogen phosphate (Na_2_HPO_4_•12H_2_O, Tianjin Hengxing Chemical Reagent Co. Ltd.). All of these reagents were of analytical grade. Tissue culture plates (Shaanxi Boda Biological Technology Co., Ltd, China) were used as the containers to place crystallisation solution.

### Solution Preparation

Four buffer systems were prepared. CH_3_COONa•H_2_O (0.2 M) and CH_3_COOH (0.3 M) were dissolved in ultrapure water to form two buffer systems (pH = 4.2, 5.4); Na_2_HPO_4_•12H_2_O (0.2 M) and NaH_2_PO_4_•2H_2_O (0.3 M) were dissolved in ultrapure water to form another two buffer systems (pH = 6.6, 7.8). NaCl was dissolved in the buffer systems at an initial concentration of 80 mg ml^−1^ to form precipitant solutions. HEWL powder was dissolved in the buffer systems at an initial concentration of 50 mg ml^−1^. Glutaraldehyde was used as the cross-linker, and it was dissolved in the precipitant solutions at volume fractions of 1%, 2%, 3%, and 4%.

### Orthogonal experiment design, protein crystallisation and chemical cross-linking by glutaraldehyde

An orthogonal array with 5 factors and 4 levels was designed in a standard L_16_ (4^5^) orthogonal array to investigate the cross-link induced cracking of HEWL crystals. The factors and corresponding levels are designed as shown in Table 3. According to the L_16_ (4^5^) orthogonal array, 16 combinations of conditions were generated. Each combination was repeated 15 times, and a statistical analysis was done to reduce the effect of experimental error on the experiment’s results.

In the experiment, the first step was growing lysozyme crystals. Crystallisation experiments were conducted by mixing equal volumes of HEWL (50 μl) and precipitant (50 μl) solution in a crystallisation plate using the sitting drop method. Then the plates were placed into a temperature controller (the temperature is highly homogeneous within ±0.1 °C in the home-made controller, whose temperature was controlled by flowing water bath through all copper walls of the cubic chamber^40^ for incubation at 11, 13, 15, or 17 °C for 2, 3, 4, 5 days according to the orthogonal array design. After incubation, the plates were taken out of the temperature controller for crystal inspection using a stereomicroscope (Olympus SZX16, Japan). The crystal images were captured by a camera (Canon DS126271, Japan). After observing the crystals, an image of a transparent sheet with an orthogonal grid as a reference was also captured at the same magnification for calculating the crystal area. It is worth mentioning that the actual area of each grid unit was 0.25 mm^2^.

After the crystals were grown, chemical cross-linking of the crystals using glutaraldehyde could be carried out. Because the remaining lysozyme molecules in the crystallisation solution would react with the cross-linker leading to formation of floccules, it is undesirable to directly carry out a cross-linking reaction with the crystals. Cross-linking of the dissolved protein in the solution would simultaneously occur, resulting in the formation of a mixture of cross-linked protein crystals and floccules that would be hard to separate from each other. To avoid this, we first removed the excess protein molecules from the solution by replacing the mother liquor with precipitant solution (100 μl) at the same precipitant concentration level. This solution exchange process was repeated twice to make sure that residual protein in solution would not interfere with the subsequent cross-linking with the lysozyme crystals. After removing the excess lysozyme molecules, we added 100 μl freshly prepared glutaraldehyde solution into the solution containing lysozyme crystals and then placed the cuvettes into the temperature controller at the designated temperature to carry out the cross-linking process. After the cross-linking process, images of the final crystals were captured using the same camera for analysis. Repeat the above steps, the 16 combinations of conditions in the orthogonal array were conducted. Furthermore, we also conducted a control experiment using aged glutaraldehyde solution in combination 2.

### Estimation of crystal area

The crystal images were two-dimensional; thus, it is hard to obtain the actual three-dimensional size (like the volume) of the crystals. However, we can use the two-dimensional area occupied by each crystal in the crystal images to roughly represent the size of the crystal for the analysis.

**Figure 6:**
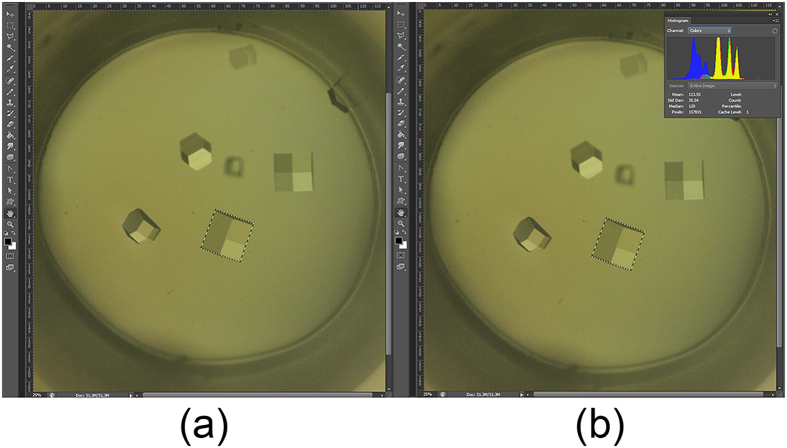
Schematic demonstration of the steps for obtaining the pixels of one crystal. (**a**) The crystal is automatically highlighted after using the “magnetic lasso” tool; (**b**) Click the “histogram” option, a new window will appear, in which the “pixel” will be shown.

To obtain the two-dimensional area of a crystal occupied in an image, we utilised the software Photoshop (version CS6; Adobe) to calculate the pixels of the crystal occupied in the picture, then with a reference picture of a grid (area of each grid unit is 0.5 mm × 0.5 mm), we can obtain the actual two dimensional area of each crystal in the captured pictures. Figure 6 shows the steps used to obtain the pixel information for a crystal from a picture. The process of obtaining the two dimensional area of a crystal (termed crystal area in this paper) is as follows: (1) Select the crystal of interest: open the image file and select the crystal using the “magnetic lasso” tool; (2) Obtain the pixel information for the selected crystal: open the menu “image” option and select the tool “histogram”. From the histogram, the pixel information for the crystal can be found; (3) Estimation of the area of the crystal in the two dimensional image: based on the following formula, the area of a crystal in the image can be estimated by equation (1):


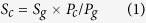


where P_c_ is the pixels of the crystal, P_g_ is the pixels of one grid unit and S_g_ is the actual area of one grid unit (i.e., 0.25 mm^2^). Repeating the aforementioned steps, we can obtain the area of every crystal in each image. After that, a statistical analysis was conducted on the area of the crystals under different combinations of preparation conditions.

## Additional Information

**How to cite this article**: Yan, E.-K. *et al*. Preparation of cross-linked hen-egg white lysozyme crystals free of cracks. *Sci. Rep.* **6**, 34770; doi: 10.1038/srep34770 (2016).

## Figures and Tables

**Table 1:** The crack ratio, and crystal area of CLPCs in an orthogonal array L_16_(4^5^).

Experimental run	Factors					Crack ratio	Crystal area
	A^a^	B^b^	C^c^	D^d^	E^e^		
1	1	1	1	1	1	0	0.0266
2	1	2	2	2	2	0.0769	0.2164
3	1	3	3	3	3	0.2541	0.1031
4	1	4	4	4	4	0.4472	0.0207
5	2	1	2	3	4	0	0.0324
6	2	2	1	4	3	0.0378	0.2541
7	2	3	4	1	2	0.3399	0.2553
8	2	4	3	2	1	0.3942	0.0289
9	3	1	3	4	2	0	0.0679
10	3	2	4	3	1	0.4148	0.2428
11	3	3	1	2	4	0.1401	0.0195
12	3	4	2	1	3	0.1841	0.0119
13	4	1	4	2	3	0	0.1074
14	4	2	3	1	4	0.3095	0.2468
15	4	3	2	4	1	0.0800	0.0230
16	4	4	1	3	2	0.2271	0.0098

^a^A represents temperature,

^b^B represents pH value,

^c^C represents crystal growth time,

^d^D represents glutaraldehyde concentration,

^e^E represents cross-linking time.

**Table 2:** Table for multi-factor analysis of variance.

Sources of variation	DEVSQ	Freedom degree	F ratio	Critical F value	Significance level
Temperature	0.004	3	1.000	F_0.1_ (3,3) = 5.39	
Solution pH	0.206	3	51.500	F_0.01_ (3,3) = 29.500	**
Crystal growth time	0.133	3	33.250	F_0.01_ (3,3) = 29.500	**
Glutaraldehyde concentration	0.020	3	5.000	F_0.1_ (3,3) = 5.39	
Cross-linking time	0.031	3	7.750	F_0.1_ (3,3) = 5.39	○

**Table 3:** The factors and levels for the orthogonal experiment design used in this research.

	Factors				
	Temperature (°C)	pH value	Crystal growth time (days)	Glutaraldehyde concentration	Cross-linking time (days)
Levels	11	4.2	2	1%	1
	13	5.4	3	2%	2
	15	6.6	4	3%	3
	17	7.8	5	4%	4
